# The accelerator, the brake, and the terrain: associations of reward-related eating, self-regulation, and the home food environment with diet quality during pregnancy and postpartum in the pregnancy eating attributes study (PEAS) cohort

**DOI:** 10.1186/s12966-020-01047-x

**Published:** 2020-11-23

**Authors:** Tonja R. Nansel, Leah M. Lipsky, Myles Faith, Aiyi Liu, Anna Maria Siega-Riz

**Affiliations:** 1 Social and Behavioral Sciences Branch, Division of Intramural Population Health Research, Eunice Kennedy Shriver National Institute of Child Health and Human Development, 6710B Rockledge Dr., MSC 7004, Bethesda, MD 20892 USA; 2 Department of Counseling, School, and Educational Psychology, Graduate School of Education, 420 Baldy Hall, University at Buffalo – SUNY, Buffalo, NY 14250-1000 USA; 3 Gillings School of Global Public Health, University of North Carolina Chapel Hill, Chapel Hill, NC 27599 USA; 4 Biostatistics and Bioinformatics Branch, Division of Intramural Population Health Research, Eunice Kennedy Shriver National Institute of Child Health and Human Development, 6710B Rockledge Dr., MSC 7004, Bethesda, MD 20892 USA; 5 Departments of Nutrition and Biostatistics and Epidemiology, School of Public Health and Health Sciences, University of Massachusetts, 109 Arnold House, 715 Pleasant St, Amherst, MA 01003-9303 USA

**Keywords:** Diet quality, Pregnancy, Postpartum, Reward-related eating, Self-regulation, Impulsivity, Delay of gratification, Home food environment

## Abstract

**Background:**

Neurobehavioral factors, including reward-related eating and self-regulation, in conjunction with the food environment, may influence dietary behaviors. However, these constructs have not been examined in pregnancy and postpartum, a time of changing appetite and eating behaviors, and when dietary intake has implications for maternal and child health. This study examined associations of reward-related eating, self-regulation, and the home food environment with pregnancy and postpartum diet quality.

**Methods:**

Participants in the Pregnancy Eating Attributes Study observational cohort were enrolled at ≤12 weeks gestation and followed through one-year postpartum. Pregnancy and postpartum Healthy Eating Index-2015 (HEI-total), and adequacy and moderation scores, respectively, were calculated by pooling 24-h diet recalls administered each trimester and during 2, 6, and 12 months postpartum. Participants completed four measures of reward-related eating – Modified Yale Food Addiction Scale (mYFAS), Power of Food Scale (PFS), Multiple Choice Procedure (MCP), and Reinforcing Value of Food Questionnaire (RVFQ); two measures of self-regulation – Barratt Impulsiveness Scale (BIS) and Delay of Gratification Inventory (DGI); and a Home Food Inventory (HFI), yielding obesogenic (OBES) and fruit/vegetables (FV) scores. Linear regression analyses estimated associations of reward-related eating, self-regulation, and home food environment with diet quality during pregnancy and postpartum, adjusting for sociodemographic characteristics.

**Results:**

Pregnancy HEI-total was inversely associated with PFS (β = − 0.14 ± 0.05, *p* = 0.009), mYFAS(β = − 0.14 ± 0.06, *p* = 0.02), 2 of the 5 RVFQ indices, MCP (β = − 0.14 ± 0.05, *p* = 0.01), and DGI food subscale (β = 0.23 ± 0.05, *p* < 0.001), but associations of postpartum HEI-total with reward-related eating measures and self-regulation were small and not statistically significant. Pregnancy and postpartum HEI-total were associated inversely with HFI-OBES (β = − 0.17 ± 0.06, *p* = 0.004 and β = − 0.19 ± 0.07, *p* = 0.006, respectively), and positively with HFI-FV (β = 0.21 ± 0.05, *p* < 0.001 and β = 0.17 ± 0.06, *p* = 0.009, respectively).

**Conclusions:**

Associations of poorer diet quality with greater reward-related eating during pregnancy but not postpartum suggests the need to better understand differences in the determinants of eating behaviors and approaches to circumvent or moderate reward-related eating to facilitate more optimal diet quality across this critical period.

**Trial registration:**

Clinicaltrials.gov. URL – Registration ID – NCT02217462. Date of registration – August 13, 2014.

## Background

Better maternal diet quality during pregnancy is related to decreased risk of excess gestational weight gain [1], gestational diabetes [2], and offspring adiposity [3]. However, across racial/ethnic groups and income levels, adherence to dietary recommendations during pregnancy is low, and little is known about influences on diet quality during this period [4–6]. Therefore, identifying modifiable determinants of diet quality in pregnancy is essential for improving numerous maternal and child health outcomes [7, 8].

Poor diet quality, in particular, excessive intake of discretionary foods (i.e., energy-dense foods providing minimal nutrient value), may result from hedonically-driven eating behavior motivated by the rewarding characteristics of food rather than homeostatic need, termed reward-related eating [9–12]. The influence of discretionary foods on brain reward circuitry [13] may additionally contribute to their preferential selection [10, 14], potentially displacing intake of more healthful foods. Previous research in non-pregnant samples using brief screeners or food frequency questionnaires has shown that reward-related eating is associated with unhealthy snack food choices in an experimental paradigm [15], and with increased intake of sugar-sweetened beverages [16], snacks [17] and discretionary foods [18–20] in observational studies. However, the relationship of reward-related eating with overall diet quality has not been examined in pregnant women, in whom preference for palatable foods may be heightened due to increased cravings [21, 22].

Reward-related eating (i.e., the “accelerator”) is hypothesized to interact with inhibitory control of eating impulses [23–25] (the “brake”) and with the availability of discretionary foods in the environment [26] (the “terrain”). Laboratory-based studies in non-pregnant samples indicate that greater self-regulation is associated with overall lower food intake [27, 28] and attenuates relationships of reward-related eating with food intake [29–31]. Additionally, a food environment abundant with highly palatable discretionary foods not only facilitates accessibility, but also increases cues prompting their intake [32]. While studies indicate that greater home food availability of discretionary foods is associated with worse diet quality in pregnant [33] and non-pregnant samples [34–38], previous studies have not investigated whether the availability of discretionary foods in the home moderates the association of reward-related eating with diet quality.

The primary aim of this study was to investigate the relationship of reward-related eating with diet quality during pregnancy and postpartum, and to examine whether self-regulation and the home food environment moderates this relationship. We hypothesized that greater reward-related eating is associated with poorer diet quality, particularly greater intake of discretionary foods, and that lower self-regulation or a more obesogenic home food environment would strengthen the relationship. Additionally, the study explores differences in measures of reward-related eating, self-regulation, and home food environment between pregnancy and postpartum.

## Methods

### Design and participants

The Pregnancy Eating Attributes Study (PEAS) was a prospective observational study of women enrolled at ≤12 weeks gestation from two university-based obstetrics clinics in Chapel Hill, North Carolina from November 2014 through October 2016 and followed through one-year postpartum [39]. The primary study aims were to examine the roles of reward-related eating, self-regulation, and home food availability on dietary intake and weight change during pregnancy and postpartum, including potential moderating roles of self-regulation and home food availability on the association of reward-related eating with diet and weight outcomes. Power analyses to determine sample size have been reported previously [39]. Data collection was completed in June 2018. Inclusion criteria were: confirmed pregnant ≤12 weeks gestation at enrollment; uncomplicated singleton pregnancy anticipated; age ≥ 18 and < 45 at screening; willingness to undergo study procedures and provide informed consent for her participation and assent for the baby’s participation; BMI ≥18.5 kg/m2; able to complete self-report assessments in English; access to Internet with email; plan to deliver at the UNC Women’s Hospital; and plan to remain in the geographical vicinity of the clinical site for 1 year following delivery. Exclusion criteria included: pre-existing diabetes; multiple pregnancy; participant-reported eating disorder; any medical condition contraindicating participation in the study such as chronic illnesses or use of medication that could affect diet or weight; or psychosocial condition contraindicating participation in the study.

### Procedures

Research staff identified potential participants through the electronic clinical appointments and medical records database. At the time of the visit, eligibility was verified and signed informed consent obtained from those electing to participate. Study visits were conducted prenatally at baseline (< 12 weeks gestation), 13–18 weeks, 16–22 weeks, and 28–32 weeks gestation, and postpartum at 4–6 weeks, 6 months, and 12 months, at which time anthropometric data and biospecimens were obtained. Self-report measures were completed online within each study visit window, prompted by email reminders to participants regarding the opening and closing of each window. Participants accessed online questionnaires and the 24-h dietary recall system through a study website developed and hosted by the study data coordinating center. Study procedures were approved by the University of North Carolina Institutional Review Board.

### Measures

#### Dietary intake

Participants were asked to complete a dietary recall within each study visit window using the Automated Self-Administered 24-Hour Recall (ASA24), a web-based tool for obtaining self-administered 24-h dietary recalls developed by the National Cancer Institute and validated against the interviewer-administered automated multiple pass method [40, 41]. The ASA24 prompts participants to indicate all foods consumed, including details of food preparation, brands, portion size, and additions. From this data, the program assigns food codes from the U.S. Department of Agriculture Food and Nutrient Database for Dietary Surveys and provide estimates of macronutrient, micronutrient, food categories and USDA Food Patterns Equivalents Database food groups. Participants received written instructions on use of the program, and research staff provided assistance if participants reported difficulty using the interface. Research staff at the University of North Carolina Nutrition and Obesity Research Core identified and corrected implausible entries (e.g., food items with implausible energy, fat or weight) and missing food or nutrient values and quantities. Dietary records indicating daily energy intakes of < 600 kcal (36 of 1883 records, 1.9%) were excluded from analyses. Dietary records with daily energy intakes of > 4500 kcal were reviewed and determined to reflect plausible intake, and thus were retained. Cutoffs for examining implausibility were based on research in non-pregnant adults indicating that energy intake cutoffs of < 500 kcal and > 3500 kcal produced similar estimates of associations of diet with BMI as compared with the Goldberg method and predicted total energy expenditure method [42]; we increased the cutoffs for this sample to account for the increased energy requirements of pregnancy [43]. Dietary intake data were used to calculate the Healthy Eating Index-2015 (HEI), an a priori indicator of diet quality that reflects conformance to the 2015 US Dietary Guidelines for Americans [44]. The HEI total score ranges from 0 to 100 and is calculated by summing 13 component scores, including 9 “adequacy components” (total fruit, whole fruit, total vegetables, greens and beans, whole grains, dairy, total protein, seafood and plant proteins, fatty acids) and 4 “moderation components” (refined grains, sodium and added sugars and saturated fats), which are calculated on a per-1000 kcal or percent of kcal basis. The 9 subscales reflecting adherence to adequacy components were summed to create a HEI-adequacy score (max score = 60), and the 4 moderation subscales were summed to create a HEI-moderation score (max score = 40). Previous studies have found little change in dietary intake across trimesters [45–47]; therefore, diet recalls from pregnancy and those from postpartum were pooled to calculate HEI across pregnancy (*n* = 365) and across postpartum (*n* = 266) using the simple HEI scoring algorithm – per person [48].

#### Hedonic hunger

The Power of Food Scale (PFS) is a 15-item questionnaire that measures hedonic hunger, the appetitive response to highly-palatable food cues in the environment [49]. Items querying response to the availability, presence, or taste of desirable food are rated on a 5-point Likert scale. The measure demonstrates strong internal consistency (Cronbach’s alpha = 0.91) and test-retest reliability (r = 0.77, *p* < 0.001), and has been validated with respect to overeating [29], outcomes of weight-loss interventions [50], and brain activity in response to viewing images of food versus control [51]. The PFS was completed each trimester during pregnancy and at 6 months postpartum (*n* = 227); mean scores across pregnancy were calculated (*n* = 377).

#### Addictive-like eating

The modified Yale Food Addiction Scale (mYFAS), a 9-item abbreviated version [52] of the Yale Food Addiction Scale assesses the presence of eating disorder symptoms consistent with diagnostic criteria for food addiction. The measure has demonstrated psychometric properties similar to the original instrument, and greater scores were associated with higher BMI across two cohorts of women [52]. The mYFAS was completed at baseline (*n* = 344) and 6-months postpartum (*n* = 217). Responses to each item were dichotomized to a score of 0 or 1 based on published cut-off values [52] and summed. Due to the highly skewed distribution, scores of 2 or more (12.5% of responses) were collapsed (only 2.3% of respondents scored 3 and 1.8% scored 4 or higher).

#### Food reinforcement measures

The Reinforcing Value of Food Questionnaire (RVFQ) [53] and Multiple Choice Procedure (MCP) [54] assessed the relative reinforcing value of food. The RVFQ asks participants to report the number of portions of a specified food that they would purchase for same-day intake at varying cost levels. The measure generates five indices: breakpoint (first price at which consumption was zero), intensity of demand (consumption at the lowest price), elasticity of demand (sensitivity of consumption to increase in cost; individual elasticities calculated using the modified exponential demand equation) [55], O_max_ (maximum expenditure), and P_max_ (price at which expenditure was maximized). The measure has demonstrated validity against a laboratory task assessing food reinforcement value [53]. The MCP asks participants to make a series of discrete choices between receiving an increasing amount of a monetary reward versus an alternative reinforcer. The datum of interest is the specific price at which participants begin to select the money over the reinforcer (breakpoint). The MCP has previously been validated in the assessment of reinforcement value of alcohol and cigarettes (e.g., [56]), and was adapted by the investigators to assess the relative reinforcing value of food. Study participants were presented with the name and images of 18 palatable foods (e.g., cookies, donuts, ice cream, chips, nachos, French fries) and asked to rate their degree of liking of each items using a labeled hedonic scale with 10 response options ranging from “most disliked sensation imaginable” to “most liked sensation imaginable” [57]. The two highest-rated foods were then used for the RVFQ and MCP, which were assessed at the first two pregnancy visits and 6 months postpartum (*n* = 209 for RVFQ and 211 for MCP). For each measure, mean scores across the two pregnancy visits were calculated (*n* = 348 for RVFQ and 350 for MCP). Due to highly skewed distributions, scores were log transformed.

#### Self-regulation

Two measures of self-regulation were administered. The 15-item short form of the Barratt Impulsiveness Scale (BIS-15) measures impulsivity across three dimensions – non-planning, motor impulsivity, and attentional impulsivity. The measure has demonstrated similar psychometric properties and associations with neurobehavioral traits as the original instrument [58]. The Delaying Gratification Inventory (DGI) is a 35-item questionnaire measuring the tendency to forego immediate satisfaction in favor of long-term rewards across five domains – food, physical pleasure, social interaction, money and achievement [59]. The subscale scores have shown good internal consistency (Cronbach’s alpha = 0.69–0.89) and strong test-retest reliability (r = 0.74–0.90). Both measures were completed at baseline (*n* = 314 for BIS and 330 for DGI) and 6 months postpartum (*n* = 215 for BIS and 219 for DGI). For this study, associations with the total score and the food subscale (DGI-food) were examined.

#### Home food environment

The Home Food Inventory includes a comprehensive range of foods in 15 categories and queries the presence of each food in the home [60]. Participants completed the inventory at baseline (*n* = 303) and 6 months postpartum (*n* = 266). Consistent with the measure’s scoring protocol, a fruit and vegetable home food environment score (HFI-FV) and an obesogenic home food environment score (HFI-OBES) were calculated as counts of the number of foods in the home in each classification. The fruit and vegetable score includes 26 common fruits and 20 common vegetables. Foods classified as obesogenic include regular-fat versions of cheese, milk, yogurt, other dairy, frozen desserts, prepared desserts, savory snacks, added fats, regular-sugar beverages, processed meat, high-fat microwavable foods, candy, and access to unhealthy foods in refrigerator and kitchen.

#### Demographic and medical characteristics

Demographic information including household composition, marital status, education, and race/ethnicity were reported by participants at baseline. Income-to-poverty ratio was calculated from participant report of total household income and household size [61]; higher values indicate greater income relative to the poverty threshold. Participant age and parity were obtained from the electronic medical record. Measured weight and height were obtained at the baseline visit and used to calculate early pregnancy BMI.

### Analysis

Paired t-tests examined differences in measures of reward-related eating, self-regulation, and home food environment between pregnancy and postpartum; Pearson correlations examined associations of these variables between pregnancy and postpartum. Multiple linear regression analyses estimated associations of measures of reward-related eating, self-regulation, and home food environment with diet quality during pregnancy and postpartum, adjusting for education, income, household size, marital status, and race/ethnicity. Postpartum analyses additionally adjusted for duration of breastfeeding. Variables were standardized prior to analyses to provide standardized estimates. Multiplicative interaction terms were used to determine whether self-regulation or home food environment moderated associations reward-related eating with diet quality. Simple slopes analyses were used to interpret significant interaction terms. Based on the findings, interactions were graphed with reward-related eating as the moderator as this provided a clearer interpretation. SPSS version 21 was used for all analyses. Analyses employed complete-case analysis; *p* values < 0.05 were interpreted as statistically significant.

## Results

**Fig. 1:**
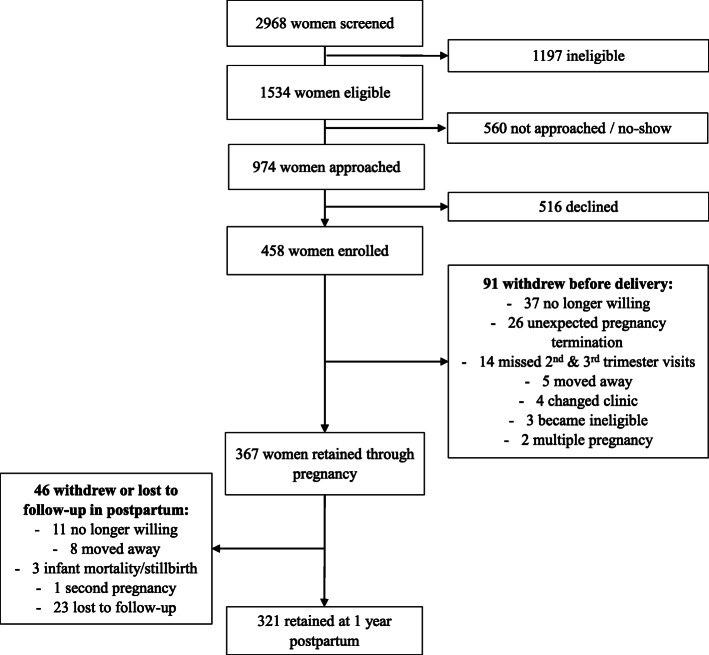
Flow of recruitment and participation in the Pregnancy Eating Attributes Study (PEAS)

Of 458 women enrolled, 91 (20%) withdrew prior to delivery and 46 (10%) withdrew during postpartum (Fig. 1). Diet records were provided by 365 participants during pregnancy and 267 during postpartum. The sample was predominantly married, college-educated, and white; approximately half were of normal weight status (Table 1). Mean HEI-total scores of ~ 58 in both pregnancy and postpartum indicate inadequate adherence to dietary guidelines. RVFQ-breakpoint, RVFQ-O_max_, and RVFQ-P_max_ were significantly lower, while BIS-15 and HFI-FV were significantly higher, during postpartum versus pregnancy (Table 2). No other measures differed significantly between pregnancy and postpartum.

**Table 1:** Sample characteristics of participants with diet recall data (*n* = 365) in the Pregnancy Eating Attributes Study (PEAS)

Demographic characteristic	Mean ± SD or N (%)
Age at baseline	30.9 ± 4.6
Household size	3.0 ± 1.2
Poverty to income ratio	3.9 ± 1.9
Marital status	
Married/living with partner	315 (92.1)
Divorced/widowed/separated/single	27 (7.9)
Education	
High school graduate or less	27 (7.9)
Some college or associate’s degree	63 (18.4)
Bachelor’s degree	106 (31.0)
Master’s/advanced degree	146 (42.7)
Race	
White	262 (75.3)
Black	49 (14.1)
Asian	17 (4.9)
Other or multi-race	20 (5.7)
Ethnicity	
Hispanic or Latino	26 (7.5)
Not Hispanic or Latino	319 (92.5)
Parity	
Nulliparous	181 (49.6)
Parous	184 (50.4)
BMI group at baseline	
Normal weight	186 (51.0)
Overweight	97 (26.6)
Obese	82 (22.5)
Healthy Eating Index total score	
Pregnancy	57.82±12.49
Postpartum	58.24±13.53

Demographic data missing for 26 participants for income; 23 participants for household size, marital status and education; 20 participants for ethnicity; and 17 participants for race

**Table 2:** Paired comparisons of reward-related eating, self-regulation, and home food environment during pregnancy and postpartum

	Pregnancy^a^	Postpartum^a^	Significance of change^b^	Correlation^c^
Power of food scale (PFS)	2.22 ± 0.61	2.25 ± 0.77	0.04 ± 0.47, t = 1.15, *p* = 0.25	0.79
Modified Yale food addiction scale (mYFAS)	0.46 ± 0.91	0.48 ± 1.01	0.03 ± 0.94, t = 0.37, *p* = 0.71	0.52
Reinforcing value of food questionnaire (RVFQ)				
RVFQ-Breakpoint	2.48 ± 0.52	2.34 ± 0.56	−0.14 ± 0.60, t = −3.35, *p* = 0.001	0.38
RVFQ-Intensity	0.64 ± 0.24	0.67 ± 0.26	0.03 ± 0.27, t = 1.39, *p* = 0.17	0.44
RVFQ-O_max_	2.36 ± 0.52	2.27 ± 0.58	−0.10 ± 0.62, t = −2.26, *p* = 0.03	0.38
RVFQ-P_max_	2.25 ± 0.47	2.08 ± 0.53	−0.17 ± 0.59, t = −3.99, *p* < 0.001	0.33
RVFQ-Elasticity	0.02 ± 0.03	0.02 ± 0.02	−0.003 ± 0.03, t = −1.6, *p* = 0.10	0.36
Multiple choice procedure (MCP)	2.27 ± 0.63	2.27 ± 0.60	0.002 ± 0.64, t = 0.05, *p* = 0.96	0.45
Barratt impulsiveness scale, short form (BIS-15)	25.30 ± 5.59	27.20 ± 5.05	1.89 ± 3.48, t = 7.67, *p* < 0.001	0.79
Delaying gratification inventory (DGI)	139.56 ± 10.18	139.13 ± 12.05	−0.42 ± 7.91, t = − 0.77, *p* = 0.44	0.76
Delaying gratification inventory, food subscale (DGI-food)	24.19 ± 3.71	24.42 ± 3.90	0.23 ± 3.37, t = 0.98, *p* = 0.33	0.61
Home food inventory, obesogenic score (HFI-OBES)	22.16 ± 8.22	22.26 ± 8.49	0.09 ± 6.98, t = 0.20, *p* = 0.84	0.61
Home food inventory, fruit and vegetable score (HFI-FV)	18.77 ± 5.46	20.17 ± 6.32	1.40 ± 5.97, t = 3.58, *p* < 0.001	0.65

^a^Values are mean ± SD. RVFQ and MCP are log-transformed

^b^Comparisons using paired-samples t-tests, with mean ± SD of change presented

^c^Pearson correlations; *p* < .001 all correlations

Pregnancy HEI-total was inversely associated with reward-related eating as measured by the PFS, mYFAS, MCP, RVFQ-intensity, and RVFQ-O_max_, but not RVFQ-breakpoint, RVFQ-P_max_, or RVFQ-elasticity (Table 3). Of these, HEI-adequacy was associated with PFS, mYFAS, RVFQ-intensity, and RVFQ-O_max_, while HEI-moderation was associated with PFS, FVFQ-intensity, and MCP. HEI-total and HEI-moderation were not associated with general measures of self-regulation but were positively associated with DGI-food; HEI-adequacy was associated with all self-regulation measures. HEI-total, adequacy, and moderation scores were associated positively with HFI-FV and inversely with HFI-OBES, except the association of HEI-moderation with HFI-OBES was at the threshold for statistical significance.

**Table 3:** Associations of reward-related eating, self-regulation, and home food environment with pregnancy diet quality

	n	HEI2015 Total Score^**a**^		HEI Adequacy Score^**a**^		HEI Moderation Score^**a**^	
		β	p	β	p	β	p
Power of food scale (PFS)	336	−0.14 ± 0.05	0.009	−0.10 ± 0.05	0.05	− 0.16 ± 0.06	0.007
Modified Yale food addiction scale (mYFAS)	311	− 0.14 ± 0.06	0.02	−0.13 ± 0.05	0.01	− 0.10 ± 0.06	0.10
Reinforcing value of food questionnaire (RVFQ)							
RVFQ-Breakpoint	320	−0.09 ± 0.05	0.11	−0.07 ± 0.05	0.13	−0.08 ± 0.06	0.19
RVFQ-Intensity	320	−0.15 ± 0.05	0.002	− 0.11 ± 0.05	0.02	−0.18 ± 0.06	0.001
RVFQ-O_max_	320	−0.12 ± 0.05	0.02	− 0.11 ± 0.05	0.03	−0.11 ± 0.06	0.05
RVFQ-P_max_	320	−0.05 ± 0.05	0.32	− 0.05 ± 0.05	0.31	−0.05 ± 0.06	0.49
RVFQ-Elasticity	320	0.03 ± 0.07	0.67	0.04 ± 0.07	0.60	0.01 ± 0.08	0.86
Multiple choice procedure (MCP)	319	−0.14 ± 0.05	0.01	− 0.07 ± 0.05	0.17	−0.21 ± 0.06	< 0.001
Barratt impulsiveness scale, short form (BIS-15)	294	−0.10 ± 0.05	0.08	− 0.11 ± 0.05	0.03	−0.04 ± 0.06	0.55
Delaying gratification inventory (DGI)	307	0.10 ± 0.05	0.08	0.11 ± 0.05	0.04	0.05 ± 0.06	0.41
Delaying gratification inventory, food subscale (DGI-food)	307	0.23 ± 0.05	< 0.001	0.19 ± 0.05	< 0.001	0.21 ± 0.06	< 0.001
Home food inventory, obesogenic score (HFI-OBES)	282	−0.17 ± 0.06	0.004	− 0.17 ± 0.06	0.003	−0.13 ± 0.07	0.05
Home food inventory, fruit and vegetable score (HFI-FV)	286	0.21 ± 0.05	< 0.001	0.18 ± 0.05	0.001	0.20 ± 0.06	< 0.001

^a^Multiple linear regression analyses; standardized coefficients adjusted for education, income, marital status, and race/ethnicity

In postpartum, diet quality was not associated with reward-related eating or self-regulation (Table 4). HEI-total, HEI-adequacy, and HEI-moderation were associated inversely with HFI-OBES and positively with HFI-FV.

**Table 4:** Associations of reward-related eating, self-regulation, and home food environment with postpartum diet quality

	n	HEI2015 Total Score^**a**^		HEI Adequacy Score^**a**^		HEI Moderation Score^**a**^	
		β	p	β	p	β	p
Power of food scale (PFS)	200	0.003 ± 0.07	0.96	0.02 ± 0.06	0.78	−0.02 ± 0.07	0.75
Modified Yale food addiction scale (mYFAS)	193	−0.04 ± 0.07	0.16	− 0.04 ± 0.07	0.61	−0.03 ± 0.08	0.74
Reinforcing value of food questionnaire (RVFQ)							
RVFQ-Breakpoint	190	0.15 ± 0.08	0.05	0.13 ± 0.07	0.08	0.15 ± 0.08	0.07
RVFQ-Intensity	190	0.01 ± 0.07	0.84	0.01 ± 0.07	0.86	0.02 ± 0.07	0.83
RVFQ-O_max_	190	0.07 ± 0.07	0.35	0.05 ± 0.07	0.53	0.09 ± 0.08	0.24
RVFQ-P_max_	190	0.10 ± 0.07	0.17	0.09 ± 0.07	0.19	0.09 ± 0.08	0.25
RVFQ-Elasticity	190	0.06 ± 0.07	0.33	0.07 ± 0.06	0.27	0.04 ± 0.07	0.58
Multiple choice procedure (MCP)	189	−0.007 ± 0.08	0.93	−0.03 ± 0.08	0.69	0.03 ± 0.08	0.68
Barratt impulsiveness scale, short form (BIS-15)	192	−0.12 ± 0.07	0.08	− 0.10 ± 0.07	0.15	−0.13 ± 0.07	0.07
Delaying gratification inventory (DGI)	195	0.14 ± 0.07	0.05	0.10 ± 0.07	0.15	0.18 ± 0.07	0.02
Delaying gratification inventory, food subscale (DGI-food)	167	0.10 ± 0.06	0.11	0.07 ± 0.06	0.28	0.14 ± 0.07	0.04
Home food inventory, obesogenic score (HFI-OBES)	225	−0.19 ± 0.07	0.006	−0.17 ± 0.07	0.01	−0.18 ± 0.07	0.01
Home food inventory, fruit and vegetable score (HFI-FV)	225	0.17 ± 0.06	0.009	0.17 ± 0.06	0.007	0.12 ± 0.07	0.07

^a^Multiple linear regression analyses; standardized coefficients adjusted for education, income, marital status, race/ethnicity, and duration of breastfeeding

**Fig. 2:**
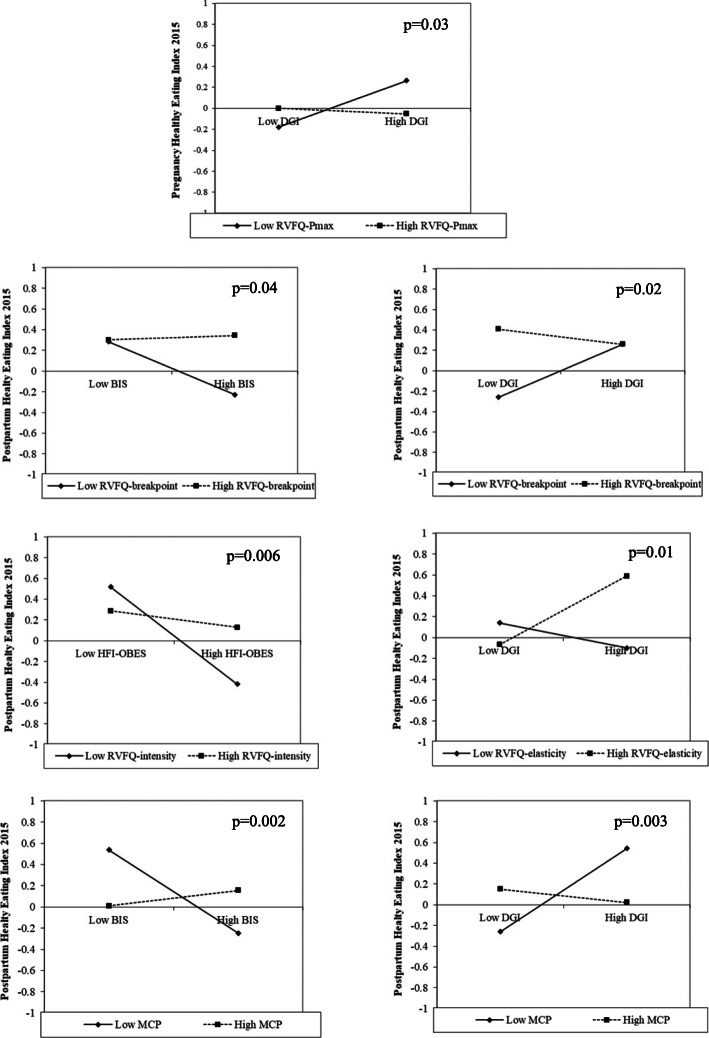
Interactions of home food environment and self-regulation with reward-related eating on diet quality. RVFQ, Reinforcing Value of Food Questionnaire; MCP, Multiple Choice Procedure; HFI-FV, fruit and vegetable home food environment score; HFI-OBES, obesogenic home food environment score; BIS-15, Barratt Impulsiveness Scale; DGI, Delaying Gratification Inventory

Tests for interaction of each measure of reward-related eating with each self-regulation and home food environment on HEI-total yielded several significant interaction terms (Fig. 2). In pregnancy, DGI interacted with RVFQ-P_max_ (β = − 0.12 ± 0.06, *p* = 0.03) such that self-regulation was positively associated with diet quality when reward-related eating was low. In postpartum, BIS (β = 0.23 ± 0.07, *p* = 0.002) and DGI (β = − 0.23 ± 0.08, *p* = 0.003) interacted with MCP; BIS (β = 0.14 ± 0.07, *p* = 0.04) and DGI (β = − 0.17 ± 0.07, *p* = 0.02) interacted with RVFQ-breakpoint, and DGI interacted with RVFQ-elasticity (β = 0.22 ± 0.09, *p* = 0.01) such that greater self-regulation was associated with higher diet quality when reward-related eating was low. Additionally, HFI-OBES significantly interacted with RVFQ-intensity (β = 0.20 ± 0.07, *p* = 0.006) such that less obesogenic home food environment was associated with higher diet quality when reward-related eating was low. No other significant interactions were observed.

## Discussion

In this sample of women followed from early pregnancy through one-year postpartum, diet quality during pregnancy was indicative of improvement needed, and was associated inversely with multiple measures of reward-related eating; however, these associations were not observed during postpartum. Additionally, pregnancy and postpartum diet quality were associated positively with fruit and vegetable availability in the home and inversely with an obesogenic home food environment.

Findings of an inverse association of reward-related eating with diet quality during pregnancy is consistent with a small body of research showing relationships of reward-related eating with greater intake of discretionary foods. Higher PFS was associated with greater intake frequency of discretionary, but not healthful, foods in US emerging adults [18] and more frequent snacking in a small sample of Australian adults [17]. Classification as “food addicted” using the YFAS was associated with greater intake of red/processed meat, low-fat snacks/desserts, and low-calorie beverages in the Nurses’ Health Study [20], greater percent energy intake from discretionary foods in Australian adults [19], and greater sweetened beverage intake in Dutch adolescents [16]. In the current study, however, associations of reward-related eating with diet quality were not more consistently observed for the moderation components than for the adequacy components, contrary to our hypothesis.

To our knowledge, no previous studies have examined reward-related eating during pregnancy and postpartum. The differences in relationships of measures of reward-related eating with diet quality between pregnancy and postpartum observed in this study were unexpected and may reflect differential influences between periods. Previous research indicates that women may relax efforts to control eating during pregnancy [62, 63], which may result in greater susceptibility to reward-related eating. Little is known about influences of eating behaviors during postpartum, when there are no physiological expectations for weight gain. Eating behaviors may be influenced by the unique demands of the postpartum period, including breastfeeding, irregular sleep, infant care, and for many, return to the workplace. Additionally, it is plausible that postpartum women may regulate their eating more carefully in an effort to return to their pre-pregnancy weight [64].

The association of higher diet quality with greater delay of gratification for food, but not overall delay of gratification, during pregnancy is a novel finding. While limited research has shown associations of general delay discounting with dietary intake [30, 65], assessment of domain-specific delay of gratification may have greater utility. Further research to determine the potential relevance of this construct and its malleability to intervention would be informative.

Consistent with previous research in pregnant [33] and general samples [34–38], better diet quality was associated with a less obesogenic home food environment. This association likely reflects both self-selection and environmental shaping. That is, food purchases likely reflect one’s eating intentions; additionally, the availability of foods in the home constrains or facilitates eating choices. In focus groups, women reported keeping foods they craved out of their homes to help them resist cravings [62]. Given that approximately two-thirds of energy intake is consumed from foods in the home [66], efforts to promote a healthful food environment during pregnancy and postpartum are likely to improve diet quality.

Findings yielded modest evidence that reward-related eating and self-regulation may interact in their influence on dietary intake; however, the interpretation of these interactions differed from that hypothesized, suggesting that reward-related eating may moderate relationships of self-regulation with dietary intake. For the significant interactions observed, differential associations were observed at low levels of reward-related eating, indicating that high reward-related eating may override the influence of self-regulation and the home food environment on diet quality. However, the interactions observed should be interpreted with caution given the multiple measures of reward-related eating and therefore large number of interaction terms tested.

Findings from this study should be interpreted in consideration of the study’s strengths and limitations. While there is no gold standard measure of reward-related eating, the use of multiple measures of the construct facilitates examination of the research question. However, measures did not include a behavioral choice task assessment or brain imaging measures of food reinforcement. Dietary intake was assessed using multiple 24-h recalls across pregnancy and postpartum; however, only one recall was obtained at each time point, precluding the ability to examine differences between trimesters or different times in postpartum. The study’s use of HEI as the outcome of interest facilitates a focus on overall dietary intake; however, it is notable that the same HEI score can be achieved through a variety of eating patterns. Additional strengths include the large sample size, repeated assessments from early in pregnancy to 1 year postpartum, and measurement of multiple potential confounders. An important limitation relates to the sample characteristics, as the study was conducted in a single geographic area with limited racial/ethnic and socioeconomic diversity; the city’s population is 73% white with a median income of $68,640, and 75% have a bachelor’s degree or higher [67]. Thus, future research examining these questions in more diverse samples is needed. Additionally, due to incomplete data in the electronic medical records, we were unable to control for smoking status. Finally, these associations are observational, and thus causality cannot be inferred. Experimental and intervention studies are needed to determine the degree to which reward-related eating and self-regulation are malleable, and whether change in these constructs and the home food environment are associated with subsequent changes in diet quality.

## Conclusion

In summary, greater reward-related eating and lower delay of gratification were associated with lower diet quality in this sample of primary well-educated women during pregnancy but not postpartum, while the home food environment was associated with diet quality across both time periods. In addition, there was some evidence that higher reward-related eating may outweigh influences of self-regulation and the home food environment. Findings suggest the importance of research to further advance our understanding of the determinants of reward-related eating and the development of approaches to circumvent or moderate them. Additionally, there is a need to better understand differences in determinants of eating behaviors between pregnancy and postpartum to facilitate more optimal diet quality during this critical period.

## Data Availability

Data described in the manuscript, code book, and analytic code will be made available upon request pending approval of a data use agreement. Following publication of study objectives, de-identified data will be shared in the NICHD Data and Specimen Hub.

## Abbreviations

HEI: Healthy Eating Index-2015
mYFAS: Modified Yale Food Addiction Scale
PFS: Power of Food Scale
MCP: Multiple Choice Procedure
RVFQ: Reinforcing Value of Food Questionnaire
HFI: Home Food Inventory
HFI-OBES: Obesogenic home food environment score
HFI-FV: Fruit and vegetable home food environment score
BIS-15: Barratt Impulsiveness Scale
DGI: Delaying Gratification Inventory
